# The Effect of Exercise on Spexin and Follistatin in Elderly Individuals

**DOI:** 10.1002/jcsm.13692

**Published:** 2025-02-02

**Authors:** Elif Yıldırım Ayaz, Berna Dincer, Gülser Cinbaz, Esra Karacan, Reyhan Kaygusuz Benli, Emel Mete, Hilal Bilgiç, Banu Mesci

**Affiliations:** ^1^ Internal Medicine Clinic, Sultan 2. Abdülhamid Han Training and Research Hospital University of Health Sciences Üsküdar İstanbul Turkey; ^2^ Department of Internal Medicine Nursing Faculty of Health Sciences, Istanbul Medeniyet University Kartal İstanbul Turkey; ^3^ Department of Physiothetapy and Rehabilitation, Health Science Faculty Yeditepe University Ataşehir İstanbul Turkey; ^4^ Department of Physiothetapy and Rehabilitation, Health Science Faculty Demiroglu Science University Şişli İstanbul Turkey; ^5^ İnternal Medicine Clinic Bagcılar Education and Research Hospital Bağcılar İstanbul Turkey; ^6^ Internal Medicine Clinic Göztepe Prof. Dr. Süleyman Yalçın City Hospital, Istanbul Medeniyet University Kadıköy İstanbul Turkey

**Keywords:** elderly individuals, exercise, follistatin, metabolic syndrome, spexin

## Abstract

**Background:**

In adipose tissue–muscle crosstalk mechanisms, the interaction of adipokines and myokines is known to be critical for maintaining the body's metabolic balance in age‐related metabolic disorders. The aim of the study investigate the effects of 12 weeks of aerobic and resistance exercise training on spexin and follistatin and their relationship with each other.

**Methods:**

This study was a multicentre, randomized controlled study conducted at two assisted living facilities with participants aged ≥ 65. Among the 66 subjects, 33 were allocated to the exercise group (E) and 33 to the control group (C). The exercise group was administered 50 min of exercise by expert physiotherapists 1 day a week for 12 weeks. Participants in the intervention groups performed exercise assignments two extra days a week, tailored to their specific circumstances and supervised by the institution's physiotherapists. Spexin, follistatin and measurements of metabolic syndrome parameters were performed at the beginning and after 12 weeks.

**Results:**

The mean age of the 62 participants who completed the study (E *n* = 31, C *n* = 31) was 73.25 ± 6.44 years, and 62.9% were female. While spexin (E = 1090.94 ± 533.66, C = 1142.91 ± 550.68 pg/mL, *p* > 0.05) and follistatin (E = 50.52 ± 24.35, C = 50.00 ± 23.52 ng/mL, *p* > 0.05) values were similar in the two groups at baseline, the values of spexin (E = 1311.32 ± 513.66, C = 1033.27 ± 486.48, *p* < 0.0001; *η* ^2^ = 0.387) and follistatin (E = 64.79 ± 32.35, C = 48.16 ± 26.27, *p* < 0.0001; *η* ^2^ = 0.267) in the exercise group were higher than in the control group at week 12. At the 12th week, neck circumference (38.32 ± 3.41, 37.16 ± 3.15, *p* = 0.002), waist circumference (102.64 ± 13.38, 98.54 ± 14.47, *p* < 0.0001), hip circumference (105.70 ± 15.43, 102.93 ± 13.48, *p* < 0.0001), body fat mass (22.69 ± 7.39, 20.45 ± 6.22, *p* < 0.0001) and systolic and diastolic blood pressure (137.19 ± 13.80, 124.9 ± 15.18, *p* = 0.0001, 77.38 ± 12.10, 72.61 ± 9.26, *p* = 0.043) decreased, and body muscle mass (46.32 ± 8.43, 49.03 ± 8.58, *p* < 0.0001) increased in the exercise group compared to baseline. A correlation was observed between the change in follistatin level and the change in spexin level (*r* = 0.438, *p* = 0.001). A negative correlation was found between the amount of decrease in body fat mass and the decrease in spexin level (*r* = −0.380, *p* = 0.005). A positive correlation was found between the increase in body muscle mass and the increase in spexin and follistatin (*r* = 0.431, *p* = 0.001; *r* = 0.490, *p* < 0.0001, respectively).

**Conclusions:**

It was found that spexin, which provides metabolic homeostasis, and follistatin, which expresses the increase in muscle mass, increased with the implementation of a 12‐week aerobic and resistance exercise program in elderly individuals, and these increases were found to be associated with each other.

**Trial Registration:**

ClinicalTrials.gov identifier: NCT05251597

## 1 Introduction

In elderly individuals, metabolic diseases tend to develop due to sedentary lifestyles, lack of balanced nutrient intake and decreased muscle mass. Exercise is an essential element in the prevention of non‐communicable metabolic diseases, which have become the pandemic of the last century, through improvements in blood glucose, blood pressure and lipid profile. Exercise interventions combining aerobic exercise and resistance training have a synergistic effect on the management of metabolic syndrome, resulting in significant improvements in muscle strength, systolic blood pressure and metabolic parameters [1].

Several mechanisms are responsible for the positive effects of exercise on metabolic health. Exerkines are humoral factors released in response to acute and/or chronic exercise that exert their exercise effects through endocrine, paracrine and/or autocrine pathways. Recent studies suggest that exerkines may have a critical role not only in the maintenance of cardiovascular and neurological health but also in the regulation of metabolic homeostasis, thereby helping to prevent metabolic diseases [2, 3]. Exerkines are highly promising in providing therapeutic modulation to ensure optimal benefit from exercise [2]. Demonstrating the effect of exercise on exerkines in elderly individuals would allow both the elucidation of exercise physiology and the development of therapeutic interventions.

Spexin (SPX) is an adipokine that regulates various metabolic processes, including metabolic stress, body weight, insulin resistance, lipid metabolism and blood pressure [4]. Spexin was found to be decreased in patients with insulin resistance, type 2 diabetes and obesity [5] and to have positive effects on adipose tissue metabolism and metabolic status [6]. It is considered that exercise may increase its release [7]. Follistatin is a myokine that stimulates muscle hypertrophy by inhibiting activin and myostatin signalling [8]. It acts as a natural antagonist of TGF‐β family members [9], slowing pancreatic β cell apoptosis [10] and maintaining glucose homeostasis. Follistatin release was reported to be increased by exercise [11].

In adipose tissue–muscle crosstalk mechanisms, the interaction of adipokines and myokines is known to be critical for maintaining the body's metabolic balance in age‐related metabolic disorders [12]. Recent pioneering studies have demonstrated the regulation of both spexin and follistatin serum levels by aerobic and resistance exercise [13, 14]. However, the relationship between the effects of exercise on spexin and follistatin is not well understood. To our knowledge, no previous study has investigated the relationship between the effects of aerobic and resistance exercise on follistatin and spexin. The present study aimed to investigate the effects of 12 weeks of aerobic and resistance exercise training on spexin and follistatin and their relationship with each other. The study hypothesized that spexin and follistatin would increase at 12 weeks after exercise training and that these increases were related. Demonstrating the relationship between the modulating effects of exercise on adipokines and myokines in elderly individuals will allow an understanding of the modifying effects of exercise at the cellular level.

## 2 Materials and Methods

### 2.1 Study Design

This multicentre, randomized controlled trial was conducted in two assisted living facilities in Istanbul between 18.02.2022 and 24.06.2022. All individuals aged 65 and over residing in the two assisted living facilities where the research was conducted were invited to the project. Written brochures and announcements were made through assisted living facility administrators. Individuals who agreed to participate in the project were evaluated for eligibility by specialist cardiology and internal medicine physicians. Individuals aged 65 years and older, with a Mini‐Mental State Examination (MMSE) score above 24 points, with adequate communication skills and who were concluded to be cardiac fit for exercise (resting electrocardiogram, questioning of cardiac symptoms and requesting further tests such as exercise electrocardiogram and myocardial perfusion scintigraphy if necessary) were included in the research. Those with uncontrolled arrhythmia or hypertension, Alzheimer's disease, Parkinson's disease, epilepsy, orthopaedic injuries that hindered walking, visual loss or hearing loss were excluded.

### 2.2 Approval of Ethical/Consent

The study was conducted in accordance with the ‘Declaration of Helsinki Ethical Principles for Medical Research Involving Human Subjects’. The approval of Istanbul Medeniyet University Prof. Dr. Suleyman Yalcin City Hospital Clinical Research Ethics Committee was obtained before the initiation of the study (approval number: 2021/0433, date: 06.10.2021). The research was carried out within the scope of the ‘Age Healthy with Green Exercise’ project implemented with the ‘Cooperation in Education Implementation Protocol’ between T.C. Uskudar Municipality and Istanbul Medeniyet University. Official permission was obtained from the Turkish Ministry of Family and Social Services to conduct the study in assisted living facilities. Participants were informed about the research objectives, procedures and data confidentiality before participating in the research, and they were explained that participation was voluntary and that they could withdraw from the research at any time. A detailed informed consent form approved by the ethics committee was signed by all participants. This randomized controlled trial adhered to the Consolidated Standards of Reporting Trials (CONSORT) reporting guideline.

### 2.3 Randomization and Blinding

**FIGURE 1:**
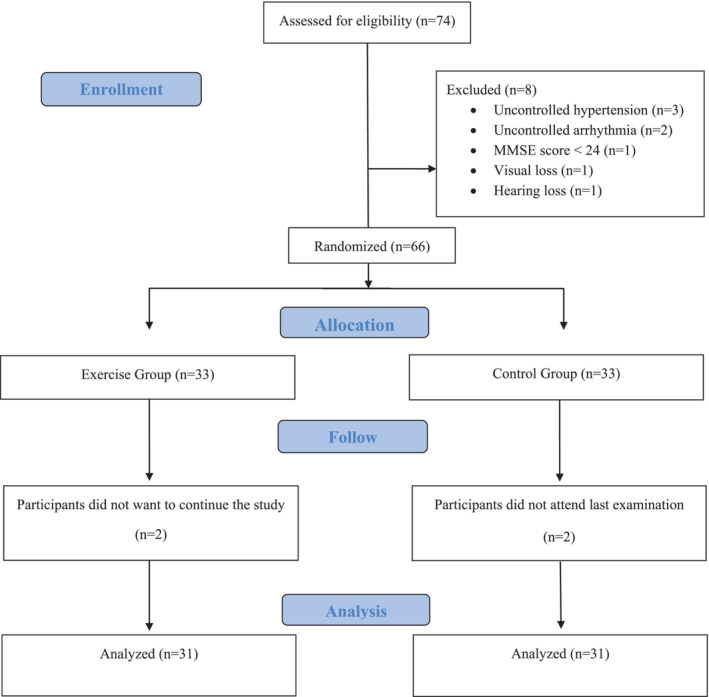
Trial flowchart.

The 74 individuals who were willing to participate in the research were evaluated. Three participants were excluded for uncontrolled hypertension, two for uncontrolled arrhythmia, 1 for MMSE score below 24, 1 for visual loss and 1 for hearing loss. Randomization of 66 participants was performed by simple numbering via the website www.randomizer.org. Of the participants, 33 were assigned to the exercise group and 33 to the control group (Figure 1). Two participants in the exercise group were excluded from the study for lack of continuation of the exercises, and 2 participants in the control group were excluded from the study for not attending the 12th‐week visit. The study was conducted with 62 participants.

Blinding was strictly enforced throughout the evaluation process to ensure objectivity and minimize bias. Specifically, the statistical analysis team was provided with data that were anonymized by replacing group names with randomly assigned numbers. This method ensured that the analysts had no knowledge of which participants belonged to which group during their analysis, thus preventing any potential bias in the statistical evaluation. Furthermore, data collection during baseline and post‐intervention were blinded, and all outcome assessors were blinded to group allocation to further enhance the study's integrity.

### 2.4 Measurements

Socio‐demographic data and comorbidity information were collected through self‐report during the initial assessment. Fasting serum collection, anthropometric, body composition and blood pressure measurements were performed at the beginning and 24 h after the last exercise session at the end of the 12‐week period. All data collection procedures were carried out by the same researchers.

### 2.5 Anthropometric and Body Composition Measurements

Body height and weight were assessed without shoes and with as little clothing as possible. Neck circumference was measured just below the larynx, and waist circumference was measured at the level of the umbilicus when standing with arms outstretched at the sides, subcostal circumference at the sides, hip circumference at the symphysis pubis in front and the most protruding part of the gluteal region in the back. Body composition was assessed using a bioimpedance body composition analyser (TANITA MC 980). Visceral adiposity index (VAI) levels were calculated using the following formula: (WC/[39.68 + (1.88 × BMI)]) × (TG/1.03) × (1.31/HDL) for males; (WC/[36.58 + (1.89 × BMI)]) × (TG/0.81) × (1.52/HDL) for females [15].

Resting blood pressure was measured twice using a semi‐automatic sphygmomanometer (Omron M4 basic digital sphygmomanometer) according to the standard procedure, and the mean of the measurements was recorded.

### 2.6 Laboratory Measurements

Venous blood samples (5 mL) were collected from the antecubital vein by a clinical laboratory scientist into serum separator tubes (SST) following an 8‐h overnight fast. The samples were delivered to the laboratory within 10–20 min. Within 40 min, the serum was separated by centrifugation at 2000–3000 RPM (revolutions per minute) for 20 min at +4°C. The serum samples were portioned and stored in eppendorf and kept at −80°C until analysed. Glucose, insulin, triglyceride, total cholesterol, high‐density lipoprotein cholesterol (HDL‐C) and low‐density lipoprotein cholesterol (LDL‐C) values of each participant at weeks 0 and 12 were measured from the serum obtained at the end of the study. Homeostasis model assessment of insulin resistance (HOMA‐IR) was calculated with the formula: glucose (mg/dL) × insulin (mU/mL)/405. Serum spexin and follistatin levels were measured with ELISA kits (Sunred).

### 2.7 Physical Activity Measurements

Considering the potential impact of physical activity levels on metabolic parameters, as well as on spexin and follistatin concentrations, the participants' baseline physical activity levels were assessed using the International Physical Activity Questionnaire‐Short Form (IPAQ‐SF). The International Physical Activity Questionnaire‐Short Form (IPAQ‐SF) comprises seven questions. It provides data on the number of days per week and the average duration per day spent in moderate and vigorous intensity activities, as well as in sedentary behaviour over the preceding 7 days. This information is used to calculate energy expenditure in metabolic equivalents (METs). The total IPAQ‐SF score is computed using the formula ‘MET level × daily activity minutes × days per week’ and is expressed as MET‐minutes per week. Calculations can be performed for walking (3.3 METs), moderate physical activity (4 METs) and vigorous physical activity (8 METs) [16]. For this study, the IPAQ total score was computed exclusively for physical activities, excluding the sitting score from the calculation.

The baseline physical activity levels of the participants were determined to be similar. Participants were requested to maintain activity diaries to document any physical activities undertaken outside of the exercise protocol specified in this study throughout the 12‐week duration.

Furthermore, data from the participants' activity diaries indicated that no additional physical activities were undertaken beyond the exercise protocol specified in the study. Consequently, physical activity level was not considered a confounding variable in the statistical analyses of the study.

### 2.8 Intervention

Before starting the exercise training program, all participants were given a 2‐h seminar covering topics such as exercise, physical activity, the effect of exercise on a healthy life and motivational strategies for sustaining exercise. The exercise group was administered 50 min of exercise by expert physiotherapists 1 day a week for 12 weeks. Participants in the intervention groups were given exercise assignments to do two additional days a week based on their exercise groups and individual circumstances. These exercise assignments were carried out by the physiotherapists of the institution. To ensure compliance with the individualized exercise program, exercise diaries were used to monitor and verify participants' compliance with prescribed exercises.

The exercise sessions took place in the gardens of assisted living facilities or national parks, on flat or gently sloping meadows. The participants' maximum heart rates were calculated with the formula = 220 − age. Ten minutes of warm‐up exercises was followed by 20 min of walking at an intensity of 50%–70% of the maximum heart rate and a target rating of 5–6 on a scale of 0 (*sitting*) to 10 (*all‐out effort*) of the level of physical exertion according to the Borg scale [17]. Aerobic exercises were performed at 50% of the maximum heart rate for the first 4 weeks, 60% of the maximum heart rate between weeks 4 and 8 and 70% of the maximum heart rate between weeks 8 and 12. If both target heart rate and target RPE could not be reached simultaneously, heart rate was the preferred indicator of intensity reached. During the aerobic exercise, heart rate was monitored with a wrist‐worn smartwatch (Xiaomi Smart Band 5) throughout the walk. After the walk, participants were made to do stretching exercises for 5 min to cool down [18]. After the walking session, the participants performed resistance exercises with free weights.

The maximum 1 repetition testing (RM) was consistent with recognized guidelines as established by Baechle et al [19]. Participants warmed up before testing, and after a 1‐min rest after the warm‐up, participants performed 8–10 repetitions with a light load (approximately 50% of estimated 1RM). After a 1‐min rest, participants increased the load (approximately 80% of estimated 1RM). They performed it through the entire range of motion. After each successful trial, the weight was increased until an unsuccessful trial. A 1‐min rest was allowed between each trial, and 1RM was achieved in 5 trials with a 5‐min rest between each trial. Upper and lower body exercises were performed alternately to promote recovery and reduce the effects of fatigue.

The intensity of the resistance exercises was determined by performing maximum repetition tests for each muscle group subjects in the resistance group performed one set of 8–12 repetitions (~10 min) of resistance exercises at low intensity (50% of one repetition maximum) and one set of 8–12 repetitions (~10 min). During resistance exercises, the perceived exertion level according to the Borg scale was four in the first 10 min and six in the last 10 min. Supervised resistance exercises performed in small groups were individualized according to the physical fitness level measured before the exercise program. These resistance exercises targeted various muscle groups, including shoulder flexors and abductors, elbow flexors and extensors, hip flexors and extensors, knee flexors and extensors and hip abductor muscle groups. Heart rate, blood pressure and saturation measurements were recorded before and after the exercise sessions. Participants were followed up during the recovery period until their vital signs returned to resting values.

Individuals in the control group did not receive any exercise‐related intervention during the study period. However, participants in the control group were allowed to engage in other physical activities on their own. To control for the confounding effects of daily physical activity, participants were asked to keep a daily activity log to record any physical activity outside the study protocol. Additionally, regular interviews were conducted to ensure accurate reporting and to monitor their overall physical activity levels throughout the study period.

No dietary intervention was applied in this study. However, in assisted living facilities, both the exercise and control groups were provided with the same meal menus, at consistent times, previously prepared by institutional dietitians, in accordance with elderly care.

### 2.9 Statistical Analysis

The study sample size was determined as at least 26 participants in each group (total sample size: 28 × 2 = 56) for 95% power (effect size: 0.26, α: 0.05) in the power analysis performed through the G*Power 3.1.9.7 program. An analysis of repeated measures of ANOVA, within–between interaction, was utilized as the statistical test for power analysis, and the effect size (effect size: 0.26) was based on the follistatin values in the study by Barzegari Marvast et al. [20]. Considering the possibility of data loss, it was planned to increase the sample size in each group by 20% and include a total of 66 (33 × 2) participants in the study.

Statistical analyses were performed using the SPSS, version 23.0 (IBM). Normality of the data was tested using the Shapiro–Wilks test. Descriptive characteristics were presented using mean and standard deviation values for normally distributed quantitative variables and median and interquartile range values for non‐normally distributed quantitative variables. Independent *t*‐test was used to determine whether difference between the groups in baseline characteristics was statistically significant for normally distributed variables, and Mann–Whitney *U* test was used for non‐normally distributed variables. Pearson chi‐square test was also used for intergroup comparison of categorical baseline variables. Analysis of two‐way repeated‐measures ANOVA was performed for repeated measurements to determine significant differences in the changes within (time effect) and between groups (group × time effect) comparisons. Partial eta squared was considered as the effect size. Spearman correlation analysis was conducted to assess the correlation between the changes in spexin and follistatin levels and anthropometric measurements compared to their baseline values at the end of the intervention. In all statistical calculations, *p* values less than 0.05 were considered significant.

## 3 Results

### 3.1 Participants' Sociodemographic Characteristics and Baseline Variables

The mean age of the participants was 73.25 ± 6.44 years, and 62.9% were female. Participants in the exercise and control groups had similar characteristics regarding age, gender, educational status and chronic diseases (Table 1) (*p* > 0.05). Anthropometric measurements, laboratory parameters, blood pressure, spexin and follistatin levels (Table 2) were similar in both groups at baseline (*p* > 0.05).

**TABLE 1:** Comparison of the basal characteristics of the groups.

	Exercise group (*N* = 31)	Control group (*N* = 31)	*p*
Age (years)			
Mean ± SD	72.74 ± 5.80	74.29 ± 7.03	0.285^a^
Gender, *N* (%)			
Female	18 (58.1)	21 (67.7)	0.430^b^
Male	13 (41.9)	10 (32.3)	
Education status, *N* (%)			
Primary school	16 (51.6)	19 (61.3)	0.255^b^
Secondary school	6 (19.4)	3 (9.7)	
High school	7 (22.5)	3 (9.7)	
University	2 (6.5)	6 (19.3)	
Diabetes, *N* (%)	4 (12.9)	8 (25.8)	0.199^b^
Hypertension, *N* (%)	17 (54.8)	20 (64.5)	0.437^b^
ASCVD, *N* (%)	14 (45.2)	16 (51.6)	0.610^b^
Congestive heart failure, *N* (%)	3 (9.7)	4 (12.9)	0.688^b^
Chronic renal failure, *N* (%)	2 (6.5)	4 (12.9)	0.390^b^
Cancer, *N* (%)	0 (0)	1 (3.22)	0.207^b^
IPAQ‐score (MET‐min/week)			
Median	537	297	0.999^c^
(Min–max)	(0–1377)	(0–1578)	

Abbreviations: ASCVD: atherosclerotic cardiovascular disease; Min: minimum; Max: maximum; SD: standard deviation.

^a^ Independent‐sample *t* test.

^b^ Pearson chi‐square test.

^c^ Mann–Whitney *U* test.

**TABLE 2:** Comparison of the metabolic parameters and anthropometric measurements of the groups at Week 0.

	Exercise group (*N* = 31)	Control group (*N* = 31)	*p*
BMI (kg/m^2^)			
Mean ± SD	27.12 ± 6.63	27.16 ± 4.05	0.304^a^
Median	25.3	27.1	
Min–max	(20.6–35.6)	(19.4–35.7)	
Neck circumference (cm)			
Mean ± SD	38.32 ± 3.41	39.93 ± 3.82	0.609^b^
Median	39	40	
Min–max	(33–46)	(31–46)	
Upper arm circumference (cm)			
Mean ± SD	30.90 ± 7.52	30.16 ± 4.20	0.572^a^
Median	30	30	
Min–max	(23–39)	(22–38)	
Waist circumference (cm)			
Mean ± SD	102.64 ± 13.38	102.03 ± 12.52	0.394^a^
Median	100	105	
Min–max	(80–19)	(60–120)	
Hip circumference (cm)			
Mean ± SD	105.70 ± 15.43	105.45 ± 9.09	0.805^a^
Median	103	103	
Min–max	(68–129)	(91–126)	
Upper leg circumference (cm)			
Mean ± SD	48 ± 6.61	46.77 ± 5.13	0.104^b^
Median	47	48	
Min–max	(39–62)	(36–55)	
Body fat mass (kg)			
Mean ± SD	22.69 ± 7.39	22.51 ± 7.42	0.921^a^
Median	20.8	20.6	
Min–max	(12.5–46.1)	(10.9–38.5)	
Body muscle mass (kg)			
Mean ± SD	46.32 ± 8.43	47.73 ± 8.77	0.443^b^
Median	46.5	49.8	
Min–max	(31.6–66.5)	(20.8–62.4)	
HbA1C (%)			
Mean ± SD	6.37 ± 1.23	6.30 ± 1.36	0.931^a^
Median	5.9	5.9	
Min–max	(5.2–10.5)	(4.9–11.2)	
HOMA‐IR			
Mean ± SD	2.94 ± 1.87	2.89 ± 2.70	0.453^a^
Median	2.13	2.09	
Min–max	(0.73–8.29)	(0.18–13.6)	
VAI			
Mean ± SD	5.10 ± 2.41	5.26 ± 3.41	0.644^a^
Median	4.62	4.2	
Min–max	(2.26–10.59)	(1.88–16.28)	
Glucose (mg/dL)			
Mean ± SD	98.31 ± 28.37	103.55 ± 39.33	0.863^a^
Median	87	87.4	
Min–max	(69–200)	(71.7–237)	
Triglyceride (mg/dL)			
Mean ± SD	136.59 ± 54.73	143.45 ± 64.52	0.829^a^
Median	128.5	126.7	
Min–max	(55.7–277)	(67.9–319)	
HDL (mg/dL)			
Mean ± SD	48.45 ± 11.32	49.08 ± 9.53	0.418^a^
Median	43.6	50.5	
Min–max	(37–85)	(32–71)	
Non‐HDL (mg/dL)			
Mean ± SD	144.16 ± 37.55	142.65 ± 41.68	0.308^b^
Median	149.6	137.15	
Min–max	(67.3–211)	(76–227.6)	
LDL (mg/dL)			
Mean ± SD	116.85 ± 37.24	113.96 ± 42.61	0.374^b^
Median	114.68	112.25	
Min–max	(38.92–195.5)	(55.92–199.96)	
Systolic blood pressure (mmHg)			
Mean ± SD	137.19 ± 13.80	138.77 ± 16.10	0.505^b^
Median	138	141	
Min–max	(112–158)	(100–159)	
Diastolic blood pressure (mmHg)			
Mean ± SD	77.38 ± 12.10	75.67 ± 8.04	0.051^b^
Median	75	75	
Min–max	(59–115)	(55–91)	
Spexin (pg/mL)			
Mean ± SD	1090.94 ± 533.66	1142.91 ± 550.68	0.742^a^
Median	932.83	936.26	
Min–max	(387.05–2881.94)	(701.68–3328.53)	
Follistatin (ng/mL)			
Mean ± SD	50.52 ± 24.35	50.00 ± 23.52	0.831^a^
Median	45.17	43.14	
Min–max	(21.87–142.95)	(27.92–145)	

Abbreviations: HDL: high density lipoprotein; HOMA‐IR: homeostasis model assessment index–insulin resistance; LDL: low density lipoprotein; max: maximum; min: minimum; SD: standard deviation; VAI: visceral adiposity index.

^a^ Mann–Whitney *U* test.

^b^ Independent‐sample *t* test.

### 3.2 Effects of Exercise Intervention on Spexin and Follistatin Levels

In the experimental (exercise) group where resistance and aerobic exercise programs were implemented, a significant increase was observed in follistatin levels (Table 3) compared to baseline values after 12 weeks of the exercise program (*p* < 0.0001; *η* ^2^ = 0.368), while no significant difference was noted in the control group (*p* > 0.05).

**TABLE 3:** Within and between groups comparisons of spexin and follistatin.

	Exercise group			Control group			
	Pre‐exercise (Week 0) Mean ± SD	Post‐exercise (Week 12)	Time effect^a^ *F*/*p*/*η* ^2^	Pre‐exercise (Week 0)	Post‐exercise (Week 12)	Time effect^a^ *F*/*p*/*η* ^2^	Group × time effect^a^ *F*/*p*/*η* ^2^
Spexin (pg/mL)	1090.94 ± 533.66	1311.32 ± 513.66	*F* = 29.28 *p* < **0.0001** *η* ^2^ = 0.365	1142.91 ± 550.68	1033.27 ± 486.48	*F* = 6.979 *p* = 0.051 *η* ^2^ = 0.120	*F* = 32.21 *p* < **0.0001** *η* ^2^ = 0.387
Follistatin (ng/mL)	50.52 ± 24.35	64.79 ± 32.35	*F* = 29.65 *p* < **0.0001** *η* ^2^ = 0.368	50.00 ± 23.52	48.16 ± 26.27	*F* = 0.473 *p* = 0.495 *η* ^2^ = 0.009	*F* = 18.53 *p* < **0.0001** *η* ^2^ = 0.267

*Note:* Statistically significant values are presented in bold for clarity.

Abbreviations: *η* ^2^: partial eta square; SD: standard deviation.

^a^ Analysis of two‐way repeated‐measures ANOVA.

There was a significant increase in spexin levels in exercise group (*p* < 0.0001), while no significant difference was noted in the control group (Table 3).

A two‐way repeated measures of ANOVA showed significant interaction effects of group × time for spexin and follistatin levels (respectively, *p* < 0.0001; *η* ^2^ = 0.387, *p* < 0.0001; *η* ^2^ = 0.267). While spexin and follistatin values were similar in the two groups at baseline, the values in the exercise group were higher than in the control group at week 12 (Table 3).

### 3.3 Effects of Exercise Intervention on Metabolic Parameters

At the end of the 12th week, systolic blood pressure was significantly decreased in exercise and control groups (*p* < 0.05); however, diastolic blood pressure was significantly decreased in only the exercise group (*p* < 0.05). There was no significant difference in HbA1C, HOMA‐IR, VAI, glucose, triglyceride, HDL, non‐HDL and LDL in both groups at the end of the 12th week compared to baseline values (*p* > 0.05) (Table 4).

**TABLE 4:** Within and between groups comparisons of metabolic parameters.

	Exercise group (*N* = 31)			Control group (*N* = 31)			Group × time effect^a^ *F*/*p*/*η* ^2^
	Pre‐exercise	Post‐exercise	Time effect^a^ *F*/*p*/*η* ^2^	Pre‐exercise	Post‐exercise	Time effect^a^ *F*/*p*/*η* ^2^	
HbA1C (%) Mean ± SD	6.37 ± 1.23	6.07 ± 1.03	*F* = 0.70 *p* = 0.404 *η* ^2^ = 0.012	6.30 ± 1.36	6.21 ± 1.48	*F* = 0.02 *p* = 0.874 *η* ^2^ < 0.0001	*F* = 0.23 *p* = 0.632 *η* ^2^ = 0.004
HOMA‐IR Mean ± SD	2.94 ± 1.87	2.53 ± 1.66	*F* = 0.67 *p* = 0.416 *η* ^2^ = 0.011	2.89 ± 2.70	2.35 ± 1.87	*F* = 0.72 *p* = 0.398 *η* ^2^ = 0.012	*F* < 0.001 *p* = 0.983 *η* ^2^ < 0.0001
VAI Mean ± SD	5.10 ± 2.41	4.37 ± 4.28	*F* = 0.73 *p* = 0.395 *η* ^2^ = 0.013	5.26 ± 3.41	4.96 ± 2.80	*F* = 0.11 *p* = 0.741 *η* ^2^ = 0.002	*F* = 0.12 *p* = 0.712 *η* ^2^ = 0.002
Glucose (mg/dL) Mean ± SD	98.31 ± 28.37	96.03 ± 25.73	*F* = 0.05 *p* = 0.809 *η* ^2^ = 0.001	103.55 ± 39.33	96.16 ± 26.98	*F* = 0.66 *p* = 0.419 *η* ^2^ = 0.011	*F* = 0.16 *p* = 0.688 *η* ^2^ = 0.003
Trygliceride (mg/dL) Mean ± SD	136.59 ± 54.73	115.92 ± 59.57	*F* = 0.78 *p* = 0.381 *η* ^2^ = 0.013	143.45 ± 64.52	141.22 ± 61.65	*F* = 1.83 *p* = 0.181 *η* ^2^ = 0.031	*F* = 0.011 *p* = 0.917 *η* ^2^ = 0.000
HDL (mg/dL) Mean ± SD	48.45 ± 11.32	51.34 ± 12.62	*F* = 0.91 *p* = 0.344 *η* ^2^ = 0.015	49.08 ± 9.53	49.19 ± 9.75	*F* = 0.05 *p* = 0.816 *η* ^2^ = 0.001	*F* = 0.70 *p* = 0.404 *η* ^2^ = 0.012
Non‐HDL (mg/dL) Mean ± SD	144.16 ± 37.55	136.1 ± 36.93	*F* = 0.62 *p* = 0.431 *η* ^2^ = 0.011	142.65 ± 41.68	131.18 ± 39.06	*F* = 1.80 *p* = 0.185 *η* ^2^ = 0.030	*F* = 0.15 *p* = 0.700 *η* ^2^ = 0.003
LDL (mg/dL) Mean ± SD	116.85 ± 37.24	112.96 ± 33.3	*F* = 0.18 *p* = 0.666 *η* ^2^ = 0.003	113.96 ± 42.61	102.87 ± 39.28	*F* = 2.03 *p* = 0.159 *η* ^2^ = 0.034	*F* = 0.49 *p* = 0.485 *η* ^2^ = 0.008
Systolic blood pressure (mmHg) Mean ± SD	137.19 ± 13.80	124.9 ± 15.18	*F* = 11.53 *p* = **0.001** *η* ^2^ = 0.161	138.77 ± 16.10	130.03 ± 17.64	*F* = 5.86 *p* = **0.018** *η* ^2^ = 0.089	*F* = 0.47 *p* = 0.494 *η* ^2^ = 0.008
Diastolic blood pressure (mmHg) Mean ± SD	77.38 ± 12.10	72.61 ± 9.26	*F* = 4.27 *p* = **0.043** *η* ^2^ = 0.066	75.67 ± 8.04	72.8 ± 9.37	*F* = 1.54 *p* = 0.219 *η* ^2^ = 0.025	*F* = 0.33 *p* = 0.562 *η* ^2^ = 0.006

*Note:* Statistically significant values are presented in bold for clarity.

Abbreviations: *η* ^2^: partial eta square; HDL: high density lipoprotein; HOMA‐IR: homeostasis model assessment index–insulin resistance; LDL: low density lipoprotein; SD: standard deviation; VAI: visceral adiposity index.

^a^ Analysis of two‐way repeated‐measures ANOVA.

In control group, two‐way repeated measures of ANOVA showed no significant interaction effects of group × time for metabolic parameters in control group (*p* > 0.05) (Table 4).

### 3.4 Effects of Exercise Intervention on Anthropometric Measurements and Body Composition

At the end of the 12th week, neck circumference, waist circumference, hip circumference and body fat mass decreased, and body muscle mass increased significantly in the exercise group compared to baseline values. (*p* < 0.05). However, no significant difference was observed in the control group (*p* > 0.05) (Table 5). There was no significant difference in BMI, upper arm and leg circumference in both groups at the end of the 12th week compared to baseline values (*p* > 0.05) (Table 5).

**TABLE 5:** Within and between groups comparisons of anthropometric measurements and body composition.

	Exercise group (*N* = 31)			Control Group(N = 31)			Group × time effect^a^ *F*/*p*/*η* ^2^
	Pre‐exercise	Post‐exercise	Time effect^a^ *F*/*p*/*η* ^2^	Pre‐exercise	Post‐exercise	Time effect^a^ *F*/*p*/*η* ^2^	
BMI (kg/m^2^) Mean *±* SD	27.12 ± 6.63	26.92 ± 6.94	*F* = 0.54 *p* = 0.462 *η* ^2^ = 0.009	27.16 ± 4.05	26.98 ± 3.72	*F* = 0.47 *p* = 0.492 *η* ^2^ = 0.008	*F* = 0.01 *p* = 0.972 *η* ^2^ = 0.000
Neck circumference (cm) Mean *±* SD	38.32 ± 3.41	37.16 ± 3.15	*F* = 10.45 *p* = **0.002** *η* ^2^ = 0.148	39.93 ± 3.82	39.18 ± 3.80	*F* = 2.33 *p* = 0.132 *η* ^2^ = 0.037	*F* = 1.45 *p* = 0.232 *η* ^2^ = 0.024
Upper arm circumference (cm) Mean *±* SD	30.90 ± 7.52	30.00 ± 5.53	*F* = 0.36 *p* = 0.547 *η* ^2^ = 0.006	30.16 ± 4.20	30.96 ± 5.60	*F* = 0.292 *p* = 0.591 *η* ^2^ = 0.005	*F* = 0.65 *p* = 0.421 *η* ^2^ = 0.011
Waist circumference (cm) Mean *±* SD	102.64 ± 13.38	98.54 ± 14.47	*F* = 17.17 *p* < **0.0001** *η* ^2^ = 0.223	102.03 ± 12.52	102.12 ± 8.92	*F* = 0.01 *p* = 0.922 *η* ^2^ < 0.0001	*F* = 8.99 *p* = **0.004** *η* ^2^ = 0.130
Hip circumference (cm) Mean *±* SD	105.70 ± 15.43	102.93 ± 13.48	*F* = 5.59 *p* = **0.021** *η* ^2^ = 0.085	105.45 ± 9.09	105.87 ± 9.66	*F* = 0.12 *p* = 0.722 *η* ^2^ = 0.002	*F* = 3.70 *p* = 0.059 *η* ^2^ = 0.058
Upper leg circumference (cm) Mean *±* SD	48.00 ± 6.61	46.32 ± 6.71	*F* = 1.30 *p* = 0.258 *η* ^2^ = 0.021	46.77 ± 5.13	47.7 ± 5.36	*F* = 0.40 *p* = 0.526 *η* ^2^ = 0.007	*F* = 1.58 *p* = 0.213 *η* ^2^ = 0.026
Body fat mass (kg) Mean *±* SD	22.69 ± 7.39	20.45 ± 6.22	*F* = 27.61 *p* < **0.0001** *η* ^2^ = 0.315	22.51 ± 7.42	22.53 ± 7.31	*F* = 0.00 *p* = 0.964 *η* ^2^ < 0.0001	*F* = 14.04 *p* < 0.0001 *η* ^2^ = 0.190
Body muscle mass (kg) Mean *±* SD	46.32 ± 8.43	49.03 ± 8.58	*F* = 23.62 *p* < **0.0001** *η* ^2^ = 0.283	47.73 ± 8.77	46.80 ± 8.74	*F* = 2.76 *p* = 0.102 *η* ^2^ = 0.044	*F* = 21.27 *p* < **0.0001** *η* ^2^ = 0.262

*Note:* Statistically significant values are presented in bold for clarity.

Abbreviations: *η* ^2^: partial eta square; SD: standard deviation.

^a^ Analysis of two‐way repeated‐measures ANOVA.

In exercise group, a two‐way repeated measures of ANOVA showed significant interaction effects of group × time for waist circumference (*p* = 0.004; *η* ^2^ = 0.130) and body muscle mass (*p* < 0.0001; *η* ^2^ = 0.262). However, BMI, body fat mass, neck, hip, upper arm and upper leg circumference showed no significant interaction effects of group × time (*p* > 0.05) (Table 5).

### 3.5 Effects of Exercise Intervention on Correlation of Spexin and Follistatin Levels

The correlation of spexin and follistatin levels with differences in body composition and anthropometric measurements from 0 to 12 weeks is given in Table 6. A moderate, negative and significant correlation was found between the amount of decrease in body fat mass and the decrease in spexin level (*r* = −0.380, *p* = 0.005). Moderate, positive and significant correlations were found between the increase in body muscle mass and the increase in spexin and follistatin levels (*r* = 0.431, *p* = 0.001; *r* = 0.490, *p* < 0.0001, respectively). A moderate, positive, significant correlation was observed between the change in follistatin level and the change in spexin level (*r* = 0.438, *p* = 0.001).

**TABLE 6:** Correlation of spexin and follistatin levels with differences in body composition and anthropometric measurements from 0 to 12 weeks.

Difference	Difference of spexin (pg/mL)		Difference of follistatin (ng/mL)	
	*r*	*p* ^a^	*r*	*p* ^a^
Body fat mass (kg)	−0.380	**0.005**	−0.182	0.193
Body muscle mass (kg)	0.431	**0.001**	0.490	**< 0.0001**
Waist circumference (cm)	−0.234	0.092	−0.223	0.108
Hip circumference (cm)	−0.271	0.050	−0.155	0.267

	Difference of follistatin (ng/mL)	
	*p* ^a^	*r*
Difference of spexin (pg/mL)	**0.001**	0.438

*Note:* Statistically significant values are presented in bold for clarity.

Abbreviation: *r*: correlation coefficient.

^a^ Spearman correlation.

## 4 Discussion

**FIGURE 2:**
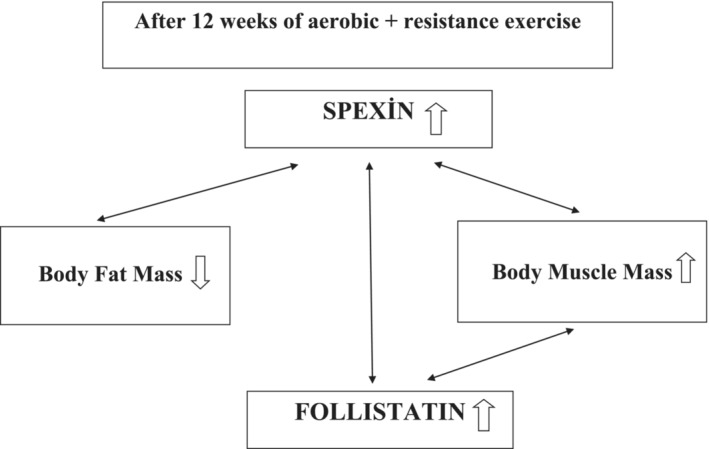
Interconnection between the spexin, follistatin, body fat and muscle mass after 12 weeks of exercise.

In this study, investigating the effects of aerobic and resistance exercise training on spexin and follistatin in elderly individuals, it was concluded that spexin, a negative regulator of metabolic stress, and follistatin, which expresses muscle hypertrophy, increased after 12 weeks of exercise. These increases were found to be correlated with each other. It was observed that neck circumference, waist circumference, hip circumference, body fat mass, systolic and diastolic blood pressure decreased and body muscle mass increased with exercise. The decrease in fat mass with exercise was associated with spexin, while the increase in muscle mass was associated with both spexin and follistatin (Figure 2).

Spexin is a 14 amino acid peptide adipokine that interferes with metabolic processes such as energy balance, fat metabolism, glucose homeostasis and weight control [5]. It is also secreted from the liver, kidney, thyroid, pancreatic islets, adrenal gland, ovary, skin, lung, stomach, small intestine, colon, oesophagus and testis in various species [21]. It provides insulin‐sensitizing and anti‐inflammatory effects and shows anorexigenic effects with hypothalamic control [22, 23]. Spexin stimulates proliferation of C2C12 cells and metabolic regulation of ERK1/2 phosphorylation in skeletal muscle via galanin receptor 2 (GALR2) and galanin receptor 3 (GALR3), G‐protein coupled receptors in the central nervous system and peripheral tissues [24, 25].

Khadir et al. found that spexin levels were lower in obese individuals compared to normal‐weight individuals, and spexin levels increased with exercise in obese individuals [26]. A significant increase in spexin in exercise‐responsive individuals (as determined by an increase in VO_2max_) was found to be an indicator of an improved metabolic profile [26]. In a 12‐week exercise study in individuals with type 2 diabetes, spexin levels increased by 66.2% with resistance exercise and 46.5% with aerobic exercise. [13]. In the present study, it increased by 30.3% with aerobic + resistance exercise. The lower rate of increase in spexin could be attributed to the older study population, which limited the exercise intensity. It was reported that performing physical exercise at a higher intensity or increasing the level of daily physical activity would affect the increase in spexin [27]. In a study evaluating the acute effects of exercise, it was observed that exercise performed both in the morning and in the evening increased serum spexin levels after exercise [28]. The data obtained in our study with elderly individuals support the literature. To our knowledge, this is the first randomized controlled trial to examine the chronic effects of exercise on spexin in elderly individuals.

Previous studies showed that physical exercise increased not only spexin secretion but also the expression of galanin and its receptors GALR2 and GALR3 in peripheral tissues [21]. In addition to increasing spexin levels, exercise is considered to provide metabolic benefits through multifaceted mechanisms by up‐regulating spexin receptor mediators. Comprehensive randomized controlled trials are required in this field.

Follistatin competitively inhibits myostatin, which inhibits muscle growth, and is also effective in promoting muscle growth independently of myostatin [29]. It regulates energy metabolism, is negatively associated with insulin resistance and has an anti‐inflammatory effect [30]. It induces browning of white adipose tissue [31], reduces ROS production [32] and leads to the inhibition of the TGF‐beta family and extracellular matrix turnover [33]. In acute exercise, glucagon synthesis from the liver increases, and insulin secretion decreases, which increases follistatin levels [34]. Elevated follistatin levels promote sustained glucose uptake by skeletal muscle, white adipose tissue lipolysis and free fatty acid uptake [35]. The metabolic benefits of elevating follistatin with acute exercise have led to questions about whether it would be elevated with chronic exercise training, promising researchers [36]. A study in older adults with sarcopenia showed that follistatin levels increased with 16 weeks of resistance exercise [37]. In a study conducted in young women, no change in follistatin gene expression was observed in muscle biopsies examined after eccentric exercise and concentric exercise [38]. This result could be attributed to the evaluation of follistatin values using muscle biopsies. The levels of follistatin, mainly synthesized in the body from the liver, were evaluated in serum in our current study and were found to increase significantly after exercise.

In this study, follistatin increase in elderly individuals after exercise was associated with an increase in body muscle mass. While this result is consistent with the described effects of follistatin, it is essential that this benefit was achieved in elderly individuals. The interaction with follistatin's inhibition of myostatin has a critical role in muscle development and muscle mass increase. Research showed that increasing follistatin levels promoted increased muscle mass by reducing the restrictive effect of myostatin on muscle growth [39].

In addition, an increase in spexin was associated with both an increase in muscle mass and a decrease in fat mass. Our study is unique in showing the relationship of exercise with these changes in muscle and fat mass. It was determined that obese children had decreased spexin levels, and spexin was negatively correlated with both insulin sensitivity and pancreatic β‐cell function [5]. Observational studies showed that spexin was associated with obesity, muscle insulin resistance and type 2 diabetes [27]. It is known that spexin increases lipolysis, decreases lipogenesis and inhibits hepatic fat accumulation [40]. The present randomized controlled trial contributes significantly to the literature by demonstrating that spexin mediates the effects of exercise on the increase in muscle mass and decrease in fat mass.

The finding that exercise is associated with an increase in spexin levels and follistatin levels supports the hypothesis that exercise may be a potential synergist for muscle‐related and metabolic health benefits in older individuals. More specifically, increased follistatin levels with exercise protect against ageing‐related muscle loss by promoting muscle anabolism, while elevated spexin levels help regulate energy homeostasis, additively affecting metabolic health in a multifaceted and positive manner. This synergistic increase also highlights the need for further research to unravel the complex molecular interactions underlying the beneficial effects of regular physical activity on ageing muscle and metabolic systems.

In this study conducted in elderly individuals, it was reported that exercise decreased neck circumference and waist circumference, which are associated with visceral adiposity [41]. Body fat mass was also found to be decreased. It was determined that there was a decrease in systolic and diastolic blood pressure. All these positive metabolic effects emphasize the importance of structured exercise programs in elderly individuals. Interestingly, a decrease in systolic blood pressure was observed in the control group. However, at the end of 12 weeks, blood pressure in the exercise group was still lower than the control group. The reason for this positive effect seen in the control group may be due to the Hawthorne effect. Participants may change their behaviour because they are aware of their involvement in the study. This may cause even participants in the control group to make healthier lifestyle choices.

Implementing structured exercise programs in assisted living facilities will contribute to community health by reducing non‐communicable diseases and their complications. The World Report on Ageing and Health published by the World Health Organization (WHO) in 2015 emphasized the need to develop interventions to support healthy ageing [42]. This project provides an example of interventions for healthy ageing, disability prevention, and improving the quality of life of older individuals in collaboration with public institutions, assisted living facilities, and health professionals.

The present study faces some limitations. First, the sample includes only two assisted living facilities in Istanbul, Turkey. This limits the generalizability of the study results. Second, the exercise program conducted by the researchers was limited to 1 day a week, and the other 2 days of exercise were assigned to the participants as homework. Although exercise logs were used to monitor compliance, the accuracy of movements and control of intensity were limited in self‐directed exercise. Third, blinding could not be applied to exercise intervention. In our study, blinding was applied only to the team performing the statistical analysis. Forth, individuals with a Mini‐Mental State Examination score above 24 points, adequate communication skills, and no visual or hearing problems were included in the study for safety purposes in this project conducted in a public institution. It is believed that more precise results could be obtained with the participation of more frail older individuals. Fifth, the 12‐week exercise duration limits the ability to observe the long‐term effects of exercise. This study was planned to be an intervention within the scope of preventive health services in healthy ageing. The inclusion of relatively healthier individuals may have led to healthy selection bias. Another significant limitation is that the strength of the relationships found was determined to be weak. This can be associated with the other limitations mentioned before. Multicentre, comparative, comprehensive and long‐term studies are required to investigate the dimensional effects of exercise on muscle and fat metabolism.

The strengths of the study are that the exercise intensity was progressively increased by taking into account the comorbid conditions of the participants and that the nutrition programs and social and physical facilities of the participants living in the same environment were similar. The study was carried out in a controlled manner under the supervision of public institutions and with a multidisciplinary approach under the supervision of doctors, physiotherapists and nurses.

This study is of importance as it is the first study in the literature to show the relationship between spexin and follistatin. The study highlighted spexin and follistatin, two important biomarkers in elucidating the pathways of adipose tissue‐muscle crosstalk mechanisms in the reduction of fat mass and increase of muscle mass with exercise. This allowed for setting new targets for the prevention of sarcopenic obesity, a crucial problem in older individuals.

In conclusion, it was found that spexin, which provides metabolic homeostasis, and follistatin, which expresses the increase in muscle mass, increased with the implementation of a 12‐week aerobic and resistance exercise program in elderly individuals, and these increases were found to be associated with each other. This study sheds light on the literature by revealing the role of spexin and follistatin in mediating the positive metabolic effects of exercise in adipose and muscle tissue cross‐talk mechanisms. Further studies with broader population‐based studies are required to investigate the association of spexin and follistatin with exercise‐metabolic health interactions in elderly individuals.

## Conflicts of Interest

The authors declare no conflicts of interest.
